# Genome and transcriptome analysis of the beet armyworm *Spodoptera exigua* reveals targets for pest control

**DOI:** 10.1093/g3journal/jkab311

**Published:** 2021-09-02

**Authors:** Sabrina Simon, Thijmen Breeschoten, Hans J Jansen, Ron P Dirks, M Eric Schranz, Vera I D Ros

**Affiliations:** 1 Biosystematics Group, Wageningen University & Research, 6708 PB Wageningen, The Netherlands; 2 Future Genomics Technologies, Leiden, The Netherlands; 3 Laboratory of Virology, Wageningen University & Research, 6708 PB Wageningen, The Netherlands

**Keywords:** Beet armyworm, *Spodoptera exigua*, whole genome, transcriptomics, gene expression, pest control

## Abstract

The genus *Spodoptera* (Lepidoptera: Noctuidae) includes some of the most infamous insect pests of cultivated plants including *Spodoptera frugiperda*, *Spodoptera litura*, and *Spodoptera exigua*. To effectively develop targeted pest control strategies for diverse *Spodoptera* species, genomic resources are highly desired. To this aim, we provide the genome assembly and developmental transcriptome comprising all major life stages of *S. exigua*, the beet armyworm. *Spodoptera exigua* is a polyphagous herbivore that can feed on > 130 host plants, including several economically important crops. The 419 Mb beet armyworm genome was sequenced from a female *S. exigua* pupa. Using a hybrid genome sequencing approach (Nanopore long-read data and Illumina short read), a high-quality genome assembly was achieved (N50 = 1.1 Mb). An official gene set (18,477 transcripts) was generated by automatic annotation and by using transcriptomic RNA-seq datasets of 18 *S. exigua* samples as supporting evidence. In-depth analyses of developmental stage-specific expression combined with gene tree analyses of identified homologous genes across Lepidoptera genomes revealed four potential genes of interest (three of them *Spodoptera*-specific) upregulated during first- and third-instar larval stages for targeted pest-outbreak management. The beet armyworm genome sequence and developmental transcriptome covering all major developmental stages provide critical insights into the biology of this devastating polyphagous insect pest species worldwide. In addition, comparative genomic analyses across Lepidoptera significantly advance our knowledge to further control other invasive *Spodoptera* species and reveals potential lineage-specific target genes for pest control strategies.

## Introduction

Analysis of genome and transcriptome data can be used to study many important questions ranging from species-specific mutations to comparative genomic evolutionary patterns. The genus *Spodoptera* is known for the high number of notorious pest species causing enormous agricultural damage resulting in economic losses worldwide, including *Spodoptera exigua*, *Spodoptera frugiperda*, and *Spodoptera litura* (Pogue 2002; Goergen *et al.* 2016; Cheng *et al.* 2017; EPPO 2017). The beet armyworm, *S. exigua* (Hübner) (Lepidoptera: Noctuidae) is a devastating polyphagous insect pest with a worldwide distribution (Mehrkhou *et al.* 2012; Fu *et al.* 2017), being able to feed on more than 130 plant species from at least 30 families including several economically important crops such as sugar beet, cotton, soybean, cabbage, maize, and tomato (Merkx-Jacques *et al.* 2008; Robinson *et al.* 2010a; Mehrkhou *et al.* 2012; Fu *et al.* 2017). *Spodoptera exigua* originated in Southern Asia and was subsequently introduced to other parts of the world including North America and Europe (Mehrkhou *et al.* 2012; Fu *et al.* 2017). It is widely distributed in the tropical and subtropical regions and migrates into more temperate regions throughout the growing season (Pogue 2002). Its long-distance migration likely played a major role in the geographic expansion of populations and its spread across the world (Fu *et al.* 2017). In temperate regions, it can be abundant in greenhouses (Smits *et al.* 1986).

Successful control of *S. exigua* is challenging due to its broad host range, rapid growth rate, its migratory dispersal and its ability to rapidly evolve resistance to pesticides (Fu *et al.* 2017; Hu *et al.* 2021; Huang *et al.* 2021). Moreover, the use of conventional chemical pesticides causes health and environmental issues and is generally less accepted (Wheeler 2002; Omkar 2016). Therefore, there is a pressing need for other, more sustainable, strategies to control *S. exigua* and other *Spodoptera* species. A promising approach includes RNA interference (RNAi)-based insect management (Burand and Hunter 2013; Scott *et al.* 2013; Renuka *et al.* 2017). One of the major challenges is to find target genes for RNAi to control specific pest species or a range of closely related pest species (Li *et al.* 2013; Bi *et al.* 2016; Tian *et al.* 2019). One way to select potential lineage-specific candidate genes is by carefully analyzing homologous relationships of genes in related species. Targeting specific gene(s) of single species using RNAi approaches could be an extremely powerful tool to diminish a specific pest outbreak without harming other (closely related) arthropod species (Price and Gatehouse 2008; Scott *et al.* 2013), which often does occur when applying general insecticides (Schulz 2004). Given the high pest potential of many *Spodoptera* species, lineage-specific genes should be identified that can be targeted during pest outbreaks. However, genomic studies have been focused mainly on *S. frugiperda* (Kakumani *et al.* 2014; Gouin *et al.* 2017), whereas other *Spodoptera* species have largely been neglected. To address this gap, we present the *S. exigua* genome assembly and official gene set (OGS).

In this study, we obtained an RNA-sequencing (RNA-seq) profile across all major life stages of *S. exigua*. We performed an in-depth analysis of gene expression patterns during the different developmental stages. We identified four candidate genes for RNAi-based pest management strategies, and additionally confirmed *Spodoptera*-specificity for three of them. Furthermore, we produced a *de novo* assembled genome draft of *S. exigua*, based on one female pupa.

## Materials and methods

### Breeding and sample collection

*Spodoptera exigua* specimens originated from a stock rearing of the Laboratory of Virology, Wageningen University & Research, which was initiated in July 2014 using pupae from a large continuous rearing, kindly provided by Andermatt Biocontrol (Switzerland). The rearing was kept on an artificial diet at 27°C with 50% relative humidity and a 14:10 h light:dark photoperiod. The artificial diet consisted of water, cornflour, agar, yeast, wheat germ, sorbic acid, methylparaben, ascorbic acid, and streptomycin sulfate. Disposable plastic trays covered with paper tissues and a lid were used as rearing containers for groups of maximum 35 larvae (for larger stages). Late fifth instars were transferred to a plastic tray containing vermiculite to facilitate pupation. Pupae were collected and transferred to cylindrical containers lined with paper sheets for egg deposition, with around 45 pupae per cylinder. Adult moths were provided with water. Collected eggs were surface sterilized with formaldehyde vapor to eliminate external microbial contamination.

High-molecular weight (HMW) chromosomal DNA was extracted from a female *S. exigua* pupa using the Qiagen Genomic-tip 100/G kit according to the manufacturer’s instructions (Qiagen, Venlo, The Netherlands). The quality of the extracted HMW DNA was analyzed on an Agilent 4200 TapeStation System using Genomic DNA ScreenTape (Agilent, Amstelveen, The Netherlands).

**Figure 1:**
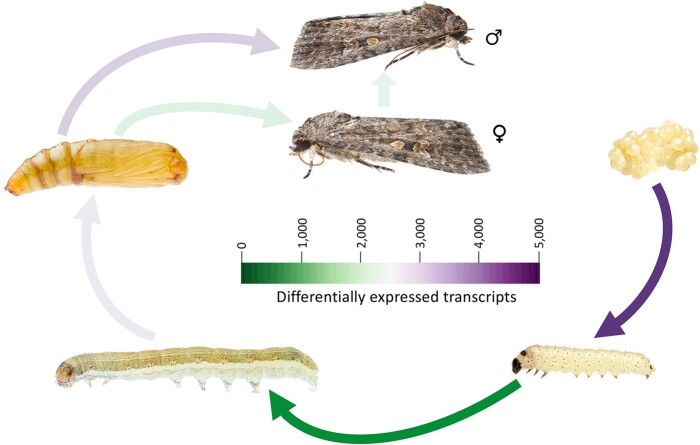
*Spodoptera exigua* life cycle and gene expression profile. The major developmental stages and sexes sequenced for *S. exigua* are shown, starting from an egg (embryonic stage) and proceeding two larval stages, namely first and third instar. After the pupal stage, there is the final differentiation into adult male and female. The color of the arrows is proportional to the number of statistically significant DE genes (FDR = 0.001, minimal fold-change of four). Note that the size of the developmental stages is not proportional.

To retrieve samples for RNA-Seq, a newly hatched male and female from the continuous rearing were mated in a plastic cup. Offspring of this couple was used for RNA-Seq, six stages were collected: embryos (eggs), first-instar larvae, third-instar larvae, pupae, male adults, female adults, with three replicates (individuals) per stage except for the embryonic stage were three clusters of each ∼100 eggs were taken. To obtain the samples, eggs were harvested, and larvae were reared as above. For the embryonic stage, egg clusters (laid within 21 h) were cut out of paper, transferred to Eppendorf tubes, snap frozen in liquid nitrogen and transferred to −80°C until shipment on dry ice to Future Genomics Technologies for further RNA extraction and sequencing. Synchronized newly hatched (non-fed) first-instar larvae, early third-instar larvae, second day pupae, and newly emerged (non-mated) female and male adults were collected. Individuals were transferred to Eppendorf tubes and snap frozen as before. For an overview of all samples please refer to Supplementary Table S1. Please also refer to Figure 1 for an overview of the developmental stages.

### Sequencing and assembly of the *Spodoptera exigua* genome

A dual sequencing approach was used for *de novo* assembly of the *S. exigua* genome sequence. In total, ∼100 Gb of raw Nanopore long-read data (Oxford Nanopore Technologies, Oxford, UK) and ∼73 Gb of raw Illumina 2 × 150 nt short-read data were generated. Long sequence read data were generated using the Oxford Nanopore Technologies platform. Prior to library preparation, HMW DNA was sheared to ∼12.5 kb fragments using Covaris gTUBE (Covaris Inc., Woburn, MA, USA). Quality was checked on the Agilent TapeStation. Library preparation was done with the SQK-LSK109 1D ligation kit from Oxford Nanopore Technologies (ONT). Samples were sequenced using one run on an ONT MinION R9.4.1 flowcell and one run on an ONT PromethION R9.4.1 flowcell, respectively. Basecalling was done with Guppy v2.2.2 (ONT MinION) and v1.6.0 (ONT PromethION), respectively. Basecalled reads were used for further processing and assembly.

In addition to long sequence read data, short-read data were generated using the Illumina NovaSeq 6000 system. Library preparation was done with the Nextera DNA Flex Library Prep Kit following manufacturers’ protocol (Illumina Inc. San Diego, CA, USA) and quality was checked using the Agilent Bioanalyzer 2100 High Sensitivity DNA Kit (Agilent, Amstelveen, The Netherlands). The genomic paired-end (PE) library was sequenced with a read length of 2 × 150 nt. Image analysis and basecalling were done by the Illumina pipeline. Please refer to Supplementary Table S2 for an overview of the DNA sequencing approach. All raw reads from the Illumina, MinION, and PromethION sequencing runs were submitted to the NCBI SRA database under accession number PRJNA623582 under sample number SAMN14550570.

To assemble the *S. exigua* genome sequence, only long sequence read data were used. First, all reads with a quality score lower than qv = 7 were removed from the long sequence read dataset. Then, the SEA program (Future Genomics Technologies BV, Leiden, The Netherlands; Jansen *et al.* 2017; program provided at the Dryad digital repository) was used to prepare seed sequences from the longest reads. In total, ∼30× estimated coverage of the longest reads was then aligned to these seeds. Reads, alignments, and seed files were used to run Tulip v. 1.0.0 (Future Genomics Technologies BV, Leiden, The Netherlands; Jansen *et al.* 2017; program provided at the Dryad digital repository) to obtain an assembly. The assembly results were used to further optimize the assembly parameters. After this optimization, the total size of the assembled genome was 419 Mb, which was divided over 946 contigs (largest contig = 4.08 Mb) with a contig N50 of 1.10 Mb. To further optimize the genome assembly, Racon (Loman *et al.* 2015) was used (two rounds) to correct mistakes in the assembly and then two rounds of Pilon polishing (Walker *et al.* 2014; Goodwin *et al.* 2015) were used to polish the assembly based on the genomic Illumina reads and to reach a high accuracy of the *de novo* assembly that was the basis for genome annotation. The final genome assembly was submitted to the NCBI GenBank database and is available under accession JACEFF000000000, version JACEFF010000000 is used in this study. As a quality check, the Benchmarking Universal Single-Copy Ortholog (BUSCO v. 3.0.2; Seppey *et al.* 2019) analysis was done on the polished *de novo* assembly using the “insecta_odb9” dataset.

### Sequencing the developmental transcriptome of *Spodoptera exigua*

Following the Illumina Truseq-stranded mRNA library prep protocol (150–750 bp inserts), we prepared 18 different indexed RNA-Seq libraries representing the different developmental stages, namely embryonic stage, early first-instar larva, early third-instar larva, pupa, adult (female and male), and including three biological replicates per stage/sex (Supplementary Table S1.1). Libraries were sequenced on an Illumina NovaSeq 6000 system at an average of 13.4 million PE2x150nt reads (6.9–22.5 million reads) per sample at Future Genomics Technologies BV, Leiden, The Netherlands. For an overview of the number of raw reads per sample please refer to Supplementary Table S1.3. The sequencing reads were quality checked using FastQC v. 0.10.1 (Andrews 2010). Adapter sequences were removed and quality-filtered using Trimmomatic v. 0.36 (Bolger *et al.* 2014), with parameters set: TruSeq3-PE-2.fa : 2:30:10, LEADING: 5, TRAILING: 5, SLIDINGWINDOW : 4:20, and removing all reads of <36 bp in length. All raw reads from the Illumina RNA-Seq approach were submitted to the NCBI SRA database under accession number PRJNA623582.

### Annotation of the *Spodoptera exigua* genome sequence

The assembled and polished genome was annotated using the maker3 pipeline (maker-3.01.02-beta). As the first step in this analysis, a repeat library was constructed with RepeatModeler (RepeatModeler-open-1.0.11; -database Spodoptera_exigua). This species-specific library was used in addition to the RepeatMasker library (Lepidoptera). For gene prediction, Augustus v. 3.3.2 was used which used the model from heliconius_melpomene1 to find genes. As additional evidence for gene models, the protein sequences for the family of the Noctuidae were extracted from UniProt (accessed March 7, 2019). Also, the RNA-Seq datasets of our 18 *S. exigua* samples were used as supporting evidence. This dataset was first assembled using the De Bruijn graph-based *de novo* assembler implemented in the CLC Genomics Workbench version 4.4.1 (CLC bio, Aarhus, Denmark). The available *S. exigua* mRNA nucleotide data from NCBI Genbank (accessed March 7, 2019) was added to this data. After running the pipeline, maker3 annotated a total of 18,477 transcripts. Gene annotations, predicted messenger RNA (mRNA) and proteins, and assemblies for gene annotations are also provided at the Dryad digital repository.

*Spodoptera exigua* proteins from the OGS v. 1.1 were further annotated using InterProScan (v. 5.36-75) with several approaches including Gene Ontology (GO) term annotation (Jones *et al.* 2014). Of the 18,477 transcripts, 16,718 transcripts retrieved annotations (Supplementary Table S3). Furthermore, the transcript OGS was used in a local BLASTX search v. 2.6.0 (Camacho *et al.* 2009; max_hsps 1, best_hit_overhang 0.1 and E-value cutoff ≤1e-3) against a locally constructed database of all Arthropoda protein sequences downloaded from the NCBI protein database (accessed, January 31, 2019). The translated proteins were additionally used in a BLASTP search v. 2.6.0 (Camacho *et al.* 2009) against the same Arthropoda database and parameters (Supplementary Tables S4 and S5).

### Transcript expression quantification

To estimate transcript expression, reads of all samples from each developmental stage were separately mapped to the newly generated *S. exigua* genome (version JACEFF010000000) using Bowtie2 v. 2.3.4 (Langmead and Salzberg 2012). The isoform and gene abundance estimations were done using RSEM v. 1.3.0 (Li and Dewey 2011). A raw (nonnormalized) count matrix was created using the perl script “abundance_estimates_to_matrix.pl” implemented in the Trinity v. 2.5.1 package (Grabherr *et al.* 2011). The count matrix was cross-sample normalized using the “calcNormFactors” function in edgeR v.3.20.8 (Robinson *et al.* 2010b; R v. 3.4.3) using trimmed mean of M values (TMM; Robinson and Oshlack 2010). See Supplementary Table S6 for the raw counts matrix of isoforms in the samples. The normalized count matrix was further filtered by abundance based on count-per-million values (CPM; to account for library size differences between samples) using edgeR v. 3.20.8 (Robinson *et al.* 2010b). Only genes with a minimum of five counts in at least two of the samples were considered expressed and retained in the dataset (see Supplementary Table S7).

To measure the similarity of the samples covering the developmental stages and to verify the biological replicates, we implemented the trinity-provided perl script “PtR.” The PCA plot is generated based on the raw nonnormalized isoform count matrix which we centered, CPM normalized, log transformed and filtered using a minimum count of 10 (Supplementary Figure S1).

The differential expression analysis was performed using DESeq2 v. 1.18.1 (Love *et al.* 2014) as implemented in the Trinity package. Transcripts were considered differentially expressed (DE) with a minimal fold-change of four between any of the treatments and a false discovery rate (FDR) of *P*-value ≤ 1e-3. The CPM and TMM normalized expression values of all DE transcripts were hierarchically clustered and cut at 50% using the Trinity-provided script “define_clusters_by_cutting_tree.pl.” This resulted in 14 clusters of DE transcripts with similar expression patterns that were used in the cluster-specific GO analysis.

**Figure 2:**
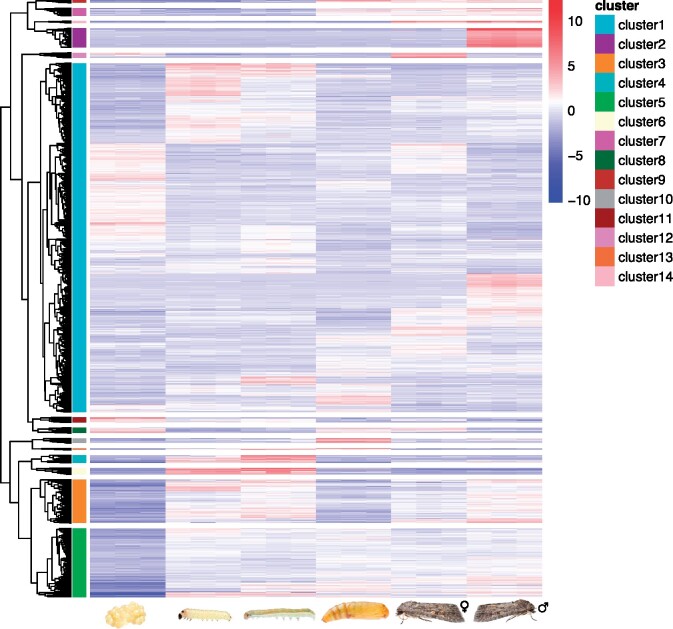
Hierarchical clustering dendrogram of all DE genes in the life cycle of *Spodoptera exigua.* Heatmap shows 9896 transcripts which have been identified DE (minimal fold-change of four, FDR ≤ 1e−3) between the six developmental stages/sexes including three replicates each (left to right: embryo, first-, third-instar larva, pupa, female adult, male adult). Transcripts from 14 distinct clusters using a cutoff at 50% (right dendrogram). The color key of the heatmap indicates low (blue) to high (red) expression values for transcripts.

See Supplementary Table S8 for an overview of cluster membership of all 9896 DE isoforms and Figure 2 and Supplementary Figure S2 for expression patterns.

**Figure 3:**
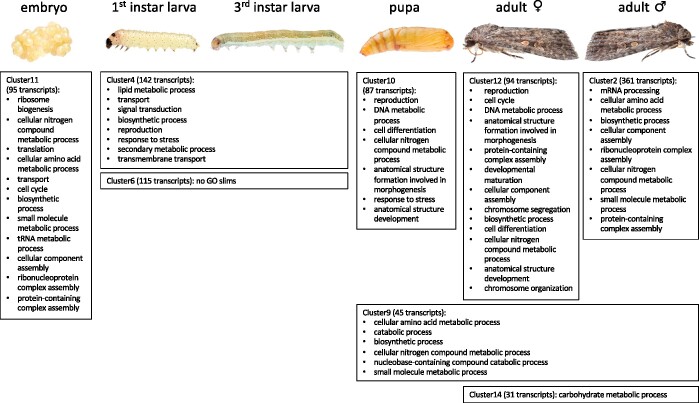
Upregulated GO slims (Biological Process) per development stages. Shown are only the eight clusters of DE transcripts that could be assigned to one developmental stage or sex or to subsequent developmental stages. The cluster number is according to the formed clusters as indicated in Figure 2. The number of transcripts is provided in parentheses as well as the statistically overrepresented GO terms (FDR ≤ 0.05) which have been summarized to generic GO slim categories.

GO analysis was performed using the GOseq package using the Trinity-provided script “runGOseq.R,” adjusting for transcript length bias in deep sequencing data (Young *et al.* 2010) and using the GO annotation retrieved from the Interpro annotation. See Supplementary Table S9 for an overview of GO annotations within the clusters. For the identified DE genes, statistically overrepresented GO terms in each cluster were identified using FDR-adjusted *P*-value (<0.05) and were further summarized to generic GO slim categories (Figure 3 and Supplementary Table S10) using the R package GOstats (Falcon and Gentleman 2007). R script for summarizing GO slim categories is provided at the Dryad digital repository.

### Phylogenomic analyses and comparative genome analyses

We used BUSCO v. 4.0.5 applying the insecta_odb10 as a reference lineage dataset (Seppey *et al.* 2019) and comprising in total 1367 BUSCOSs, to extract single copy complete BUSCOs on the amino acid (aa) level for *S. exigua* and another 36 lepidopteran genomes (Supplementary Table S11).

For the phylogenomic analysis, first, aa sequences of single-copy BUSCO genes were separately aligned using MAFFT v. 7.305 (Katoh and Standley 2013) using the L-INS-i algorithm. For the identification of putative ambiguously aligned or randomized multiple sequence alignment (MSA) sections, we used Aliscore v. 1.2 (Misof and Misof 2009; Kück *et al.* 2010) on each MSA with the default sliding window size, the maximal number of pairwise sequence comparisons and a special scoring for gap-rich aa data (options -r and -e). After exclusion of the identified putative ambiguously aligned or randomized MSA sections with ALICUT v. 2.3 (Kück *et al.* 2010), the final MSAs were concatenated into supermatrices using FASconCAT-G v. 1.02 (Kück and Longo 2014). The resulting dataset comprised 1367 gene partitions and 687,494 aa positions.

Prior to the tree reconstruction, the best scoring aa substitution matrix for each gene partition was selected with ModelFinder as implemented in IQ-TREE v. 1.6.12 (Kalyaanamoorthy *et al.* 2017). We restricted the search of the best fitting model to eight aa substitution matrices appropriate for nuclear markers: DCMut (Kosiol and Goldman 2005), JTT (Jones *et al.* 1992), LG (Le and Gascuel 2008), Poisson, PMB (Veerassamy *et al.* 2003), VT (Muller and Vingron 2000), and WAG (Whelan and Goldman 2001). We additionally included the protein mixture model LG4X (Le *et al.* 2012), which accounts for FreeRate heterogeneity. Furthermore, we allowed testing the default rate heterogeneity types (E, I, G, I + G, and FreeRates: R; Gu *et al.* 1995; Soubrier *et al.* 2012; Yang 1994), with or without empirical rates (-F, -FU) as well as testing the number of rate categories (-cmin 4 -cmax 15). The best model for each gene partition was selected according to the best second-order or corrected Akaike Information Criterion score (Hurvich and Tsai 1989). Dataset and partition scheme including selected models are provided at the Dryad digital repository.

Phylogenetic relationships were inferred under the maximum likelihood (ML) optimality criterion as implemented in IQ-TREE v. 1.6.12 (Chernomor *et al.* 2016; Nguyen *et al.* 2015) using the best scoring aa substitution matrix for each gene partition and the edge-proportional partition model allowing partitions to have different evolutionary rates (option -ssp). We performed 50 independent tree searches (25 searches with a random and 25 with a parsimony start tree). The resulting number of unique tree topologies was assessed with Unique Tree v. 1.9, kindly provided by Thomas Wong and available upon request. Node support was estimated via nonparametric bootstrapping of 100 bootstraps replicates in IQ-TREE and mapped onto the ML tree with the best log-likelihood.

**Figure 4:**
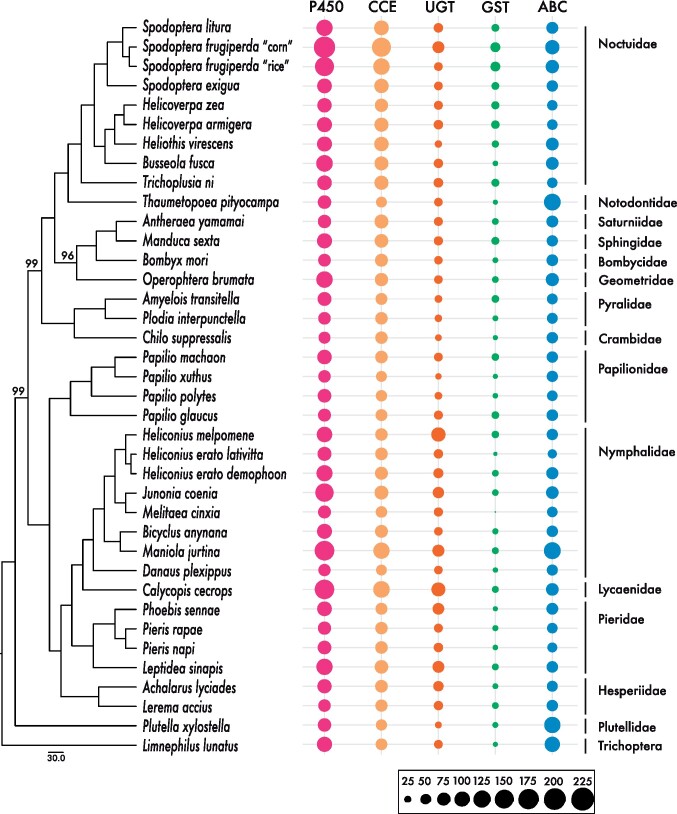
Comparison of Lepidoptera genomes and inferred phylogenetic relationships. Shown is the ML phylogeny based on 1367 single-copy BUSCOs (left, all nodes have 100% support unless otherwise noted). A number of detoxification gene members of five main detoxification families, P450s, CCEs, UGTs, GSTs, ABCs, are presented per species in a bubble plot generated with ggplot2.

We further scanned all these lepidopteran protein sets for several gene families associated with detoxification function, namely P450 monooxygenases (P450s), carboxyl- and choline esterases (CCEs), UDP-glycosyltransferases (UGTs), glutathione S-transferases (GSTs), ATP-binding cassettes (ABCs). We identified the protein families of all proteins by running InterProScan v. 5.36-75 (-appl Pfam –goterms; Jones *et al.* 2014); additionally, we ran a local BLASTP against the UniRef50 database (ftp.uniprot.org/pub/databases/uniprot/uniref/uniref50/uniref50.fasta.gz; release version July 31, 2019, accessed August 20, 2019) using an e-value cutoff of 1e-3. Based on these annotations, genes were selected to belong to any of the gene families of interest if it had a match to one of the Uniref50 cluster terms or Pfam- or InterProScan identifiers (Supplementary Table S12). The number of detoxification gene members of the five main detoxification families was plotted for each species in a bubble plot generated with ggplot2 (Wickham 2016; Figure 4).

### Comparative analysis of *Spodoptera*-specific genes

We used OrthoFinder v. 2.3.11 using default settings (Emms and Kelly 2015) to identify homologs within the *Spodoptera* clade. We included the genome protein sequence files from three *Spodoptera* species: *S. exigua* (this study), *S. litura* [direct receival OGSv1 September 28, 2019 from authors (Cheng *et al.* 2017)] and *S. frugiperda* (ftp://ftp.cngb.org/pub/CNSA/CNP0000513/CNS0099235/CNA0003276/Sf_20190612ynM_v1.pep, accessed September 20, 2019; Liu *et al.* 2019). In addition, we included five closely related but diverse Lepidoptera species: *Heliothis virescens* (ftp://ftp.ncbi.nlm.nih.gov/genomes/all/GCA/002/382/865/GCA_002382865.1_K63_refined_pacbio/GCA_002382865.1_K63_refined_pacbio_protein.faa.gz, accessed September 20, 2019; Fritz *et al.* 2018), *Helicoverpa zea* (https://data.csiro.au/collections/#collection/CIcsiro:23812v3, accessed August 21, 2019; Pearce *et al.* 2017), *Helicoverpa armigera* (https://data.csiro.au/collections/#collection/CIcsiro:23812v3, accessed August 21, 2019; Pearce *et al.* 2017), *Trichoplusia ni* (ftp://www.tnibase.org/pub/tni/tni_protein_v1.fa.gz, accessed September 20, 2019; Chen *et al.* 2019), and *Bombyx mori* (http://silkbase.ab.a.u-tokyo.ac.jp/cgi-bin/download.cgi, accessed August 20, 2019; International Silkworm Genome Consortium 2008).

We identified 119 orthogroups (OGs) containing sequences only from the 3 *Spodoptera* species (Supplementary Table S13.1). Of these 119 OGs, only 7 OGs were DE in the larval stage (cluster 4, Supplementary Table S13.2). Of these seven OGs, three OGs were “uncharacterized” protein, and four OGS were annotated as: nuclear complex protein (OG0013351), REPAT46 (OG0014254), trypsin alkaline-c type protein (OG0014208), and mg7 (OG0014260; Supplementary Table S13.2) for which we performed gene tree analyses. For the gene tree analyses, we extended our dataset based on the original OrthoFinder run by including similar sequences from related species to additionally verify the lineage-specificity of these genes. Using the identified *S. exigua* sequences within the lineage-specific OGs as queries, we searched for close homologs using BLASTX (Bravo *et al.* 2019) against the NCBI protein database online (Sayers *et al.* 2020). Thus, the resulting datasets used to construct gene trees were compiled with some differences. The gene tree of nuclear pore complex proteins was composed of *Spodoptera* OG sequences and all Lepidoptera nuclear complex DDB_G0274915 proteins from the NCBI-nr database (accessed October 2, 2020, keyword “DDB_G0274915”). The initial BLAST identifications of *Spodoptera*-specific OG sequences showed high similarity with DDB_G0274915-like nuclear pore complex proteins. For the remaining three datasets, we additionally included clusters of homologous genes from OrthoDB v. 10 (Kriventseva *et al.* 2019). For the REPAT protein dataset, we added the ortholog cluster (“16151at7088”) consisting of Multiprotein bridge factor 2 (MBF2) orthologs. MBF2 proteins are described to be homologs of REPAT genes in other Lepidoptera species, and have been therefore included (Navarro-Cerrillo *et al.* 2013). The REPAT protein gene tree dataset included all protein sequences from Navarro-Cerrillo *et al.* (2013). For a second REPAT tree, we only analyzed sequences from the βREPAT class (Navarro-Cerrillo *et al.* 2013). For both, the trypsin and mg7 gene tree datasets, we included clusters of homologous genes from OrthoDB v. 10 based on the linked cluster to our closest BLAST hit via the online NCBI protein database. For the trypsin gene tree dataset, we added the ortholog cluster “118933at50557” consisting of “serine protease” orthologs. These homologous sequences were selected because the *S. litura* sequence (“SWUSl0076430”) from the *Spodoptera*-specific OG formed a member of this group. All insect orthologs were included. Finally, the mg7 gene tree dataset included the ortholog group “15970at7088” from OrthoDB v. 10 (accessed September 15, 2020), because the *S. litura* sequence (“SWUSl0113290”) was an ortholog member. For a second tree, we included all genes derived from He *et al.* (2012), where the expression of mg7 in the midgut of *S. litura* was studied and homologs in related lepidopteran species were analyzed. Finally, we searched for potential paralogs of all target genes in the protein sets of *S. exigua*, *S. litura*, and *S. frugiperda* using BLASTP (max_hsps 1, best_hit_overhang 0.1 and E-value cutoff ≤1e-5) with NCBI-BLAST+ v. 2.6.0 (Camacho *et al.* 2009) against a local BlastDB of above gene tree datasets of nuclear pore complex, REPAT, trypsin, and mg7 proteins.

For all genes, sequences were aligned using MAFFT v. 7.471 with the L-INS-i method and default settings (Katoh and Standley 2013). Gene trees were reconstructed using IQ-TREE v. 1.6.12 (Nguyen *et al.* 2015; Chernomor *et al.* 2016) using the ML method and implementing bootstrap with 100 replications. The preferred model was applied based on the model selection (Kalyaanamoorthy *et al.* 2017). For the nuclear pore complex gene tree, the best-fit model was “WAG+F+G4,” for REPAT including both αREPAT and βREPAT proteins “WAG+F+R4,” for the gene tree consisting only βREPAT proteins “VT+G4,” for the trypsin gene tree “WAG+F+R5” finally for both mg7 based gene trees “LG+G4.” All gene alignment files are provided at the Dryad digital repository.

The gene trees were rooted dependent on included species and gene composition, aiming for earliest branching genes or species, for example, by selecting the earliest branching lineages from Kawahara *et al.* (2019). For the nuclear pore complex protein gene tree, *Papilio xuthus* was used for rooting since it branched early within Papilionidae (Kawahara *et al.* 2019). For the REPAT gene tree, we used the same approach as Navarro-Cerrillo *et al.* (2013), which rooted the tree using the REPAT-like27 and REPAT-like28 cluster. However, for the limited REPAT gene tree only including βREPAT class genes, we rooted using group V of the βREPAT class according to the first group branching off (Navarro-Cerrillo *et al.* 2013). The trypsin tree was rooted using the branch, giving rise to a Hymenoptera-specific cluster. Finally, the mg7 gene trees were rooted using either *Choristoneura fumiferana* (Tortricidae) (mg9 cluster) or, if absent, *Amyelois* (Pyralidae; Kawahara *et al.* 2019).

## Results

### Genome annotation and comparison to other Lepidoptera genomes

The total size of the final polished assembled genome was 419 Mb, which was divided over 946 contigs (largest contig = 4.15 Mb) with N50 = 1.1 Mb (Table 1). To confirm the assembly genome size, a k-mer counting approach was used. After counting the 21 and 27 mers in the Illumina dataset, the count tables were analyzed with GenomeScope. The genome size as estimated by k-mer counting was ∼370 Mb, which correlated with the Nanopore assembly size (which is slightly larger). The genome size of *S. exigua* presented here, as well as the GC content (given in %), is comparable with other published *Spodoptera* genomes and the preprint version of the *S. exigua* genome (Zhang *et al.* 2020; Table 1).

**Table 1:** Genome metrics of *Spodoptera exigua* and other published *Spodoptera* genomes

		Sequencing information			Genome assembly				
Species		Notes	Method	Reference	Genome assembly total length	Contig N50	No. of contigs	GC content	Proteins
*Spodoptera exigua*		Female pupa	Nanopore + Illumina	This study	419.3 MB	1.1 Mb	946	36.52	18,477
*Spodoptera exigua*		Female pupa	PacBio + Illumina + Hi-C	Zhang *et al.* (2020)	446.8 Mb	3.5 Mb	667	36.67	17,727
*Spodoptera litura*		Male adults	Illumina	Cheng *et al.* (2017)	438.3 Mb	0.068 Mb	13,636	37	15,317
*Spodoptera frugiperda*		Sf21 cell line	Illumina	Kakumani *et al.* (2014)	358.0 Mb	0.008 Mb	97,607	32.97	11,595
*Spodoptera frugiperda*	“corn”	Two male larvae	Illumina	Gouin *et al.* (2017)	437.9 Mb	21.6 Kb	–	36	21,700
*Spodoptera frugiperda*	“rice”	Single male larva	Illumina	Gouin *et al.* (2017)	371.0 Mb	25.4 Kb	–	36	26,329
*Spodoptera frugiperda*		Sf9 cell line	PacBio	Nandakumar *et al.* (2017)	451.0 Mb	0.25 Mb	4,577	36.53	25,699
*Spodoptera frugiperda*	“corn”	Two male larvae	PacBio + Illumina + Hi-C	Gimenez *et al.* (2020), Nam *et al.* (2020)	384.4 Mb	—	—	36.34	21,839
*Spodoptera frugiperda*	“rice”	Single male larva	PacBio + Illumina + Hi-C	Gimenez *et al.* (2020), Nam *et al.* (2020)	379.9 Mb	—	—	36.37	22,026
*Spodoptera frugiperda*		Single male adult	MGISEQ + Hi-C	Gui *et al.* (2020)	543.7 Mb	0.09 Mb	29,584	36.52	22,201
*Spodoptera frugiperda*		Single male adult	PacBio + Illumina + Hi-C	Zhang *et al.* (2020)	390.4 Mb	5.6 Mb	776	36.4	22,260
*Spodoptera frugiperda*		Female pupa	PacBio + Hi-C	Xiao *et al*. (2020)	486.3 Mb	1.1 Mb	618	36.4	22,623

The BUSCO (v. 3) assessments indicated that the quality and completeness of our *de novo* assembly was good (complete: 96.8%; fragmented: 1.0%; missing: 2.2%) and comparable with other lepidopteran genomes (Supplementary Figure S3). By these quality metrics, the *S. exigua* assembly is comparable with those of fellow lepidopterans, facilitating comparative genomic analyses.

Using our final assembly, an OGS was generated by automatic annotation and transcriptomic RNA-seq datasets of 18 *S. exigua* samples (see below) as supporting evidence. The OGS (v. 1.1), consists of 18,477 proteins and is provided at the Dryad digital repository.

### Gene expression analyses across the whole life-cycle of *Spodoptera exigua*

The major developmental stages across the whole life-cycle of *S. exigua*, namely embryonic stage (egg), early first-instar larva, early third-instar larva, pupa, and adult (both sexes: female and male), were sequenced on an Illumina NovaSeq 6000 system at an average of 13.4 million PE2x150nt reads (6.9–22.5 million reads per sample; Supplementary Table S1.3). Based on these reads, we performed differential expression analyses using our *de novo* assembled *S. exigua* genome as a reference.

We first compared gene expression from subsequent different developmental stages and sexes based on pairwise comparisons to determine the dynamic changes in gene expression during development. A striking number of significantly DE transcripts (*n* = 4974 transcripts) was detected during early embryonic development (between the embryonic and the first-instar larval stage; Figure 1). Notably, this rapid change in the expression dynamics of *S. exigua* was the largest during the entire life cycle (Figure 1 and Supplementary Table S14). In contrast, the smallest change in gene expression was between first- and third-instar larvae (*n* = 1222 transcripts). A larger change in gene expression was also observed between pupa and male adult (*n* = 3112 transcripts) compared with pupa to female adult (*n* = 2061 transcripts), likely due to the fact that female pupae were analyzed. For an overview of relationships between the different life stages based on identified significant changes in gene expression see Supplementary Figure S4. Supplementary Table S15 provides an overview of all DE genes identified per pairwise comparison of the developmental stages.

We further identified 9896 transcripts as DE across all pairwise comparisons. Hierarchical clustering revealed 14 clusters of DE transcripts with similar expression patterns (Figure 2). Of these, the gene expression of eight clusters could be associated with a single developmental stage or similar subsequent developmental stages, for example, one cluster for the larval stage (see also Supplementary Figure S2). For these eight clusters, statistically overrepresented GO terms were identified using FDR-adjusted *P*-value (<0.05) and were further summarized to generic GO slim categories (Figure 3).

For the embryonic stage (cluster 11, Figure 3), there was an enrichment of GO categories associated with ribosome biogenesis (GO:0042254), ribonucleoprotein complex assembly (GO:0022618), transfer RNA (tRNA) metabolic process (GO:0006399), translation (GO:0006412), and cell cycle (GO:0007049). The enrichment of these categories highlights the rapid succession of cell cycles associated with chromatin replication and initiation of transcription and translation for embryo patterning (Koutsos *et al.* 2007). Detailed investigation of DEs gene annotations based on the Arthropoda database (Supplementary Tables S4 and S5) revealed several known genes important in morphogenesis, for example, during the embryonic stage Krüppel-like transcription factors (Kaczynski *et al.* 2003; McCulloch and Koenig 2020), specificity proteins (Kennedy *et al.* 2016), and several WD-repeat containing proteins (Smith 2008).

We did not identify a specific cluster for the first larval stage nor for the third larval stage, but rather one cluster including both larval stages (=larval stage cluster, cluster 4, Figure 3). The larval stage was enriched for genes involved in general metabolic processes, such as signal transduction (GO:0007165), biosynthetic processes (GO:0009058), and secondary metabolic processes (GO:0019748). Several genes having a key role in the digestion of plant material and herbivore success were significantly DE within the larval stage (see Supplementary Table S4). These include REPAT genes (Herrero *et al.* 2007; Navarro-Cerrillo *et al.* 2013), trypsins (Muhlia-Almazán *et al.* 2008), cuticle proteins (Celorio‐Mancera *et al.* 2013; Müller *et al.* 2017; Orsucci *et al.* 2018; Breeschoten *et al.* 2019), and members of prominent detoxification gene families such as cytochrome P450s (P450), carboxyl/cholinesterases (CCEs), GST, and UGT. The pupal stage varied from the larval stage in that there was significant enrichment in processes associated with cell differentiation (GO:030154), anatomical structure formation involved in morphogenesis (GO:0048646), and anatomical structure development (GO:0048856).

We further identified several pupal cuticle proteins as significantly DE within this pupal stage. The female adult stage (cluster 12) was enriched for genes involved in for example, cell cycle (GO:0007049), chromosome segregation (GO:0007059) and chromosome organization (GO:0051276), anatomical structure development (GO:0048856), and biosynthetic process (GO:0009058) and we identified orthologs of several homeotic genes(-like), such as *Bicaudal C*, *Sex combs reduced*, and *proboscipedia*. For the male adult stage (cluster 2, Figure 3), there was an enrichment of GO categories associated with for example, mRNA processing (GO:0006397), cellular aa metabolic process (GO:0006520), cellular component assembly (GO:0022607), and biosynthetic process (GO:0009058). For the female and the male adult stage, we further identified several sex-specific genes as DE, such as vitellogenin and vitellogenin receptor in the female (Rotllant *et al.* 2017) and testis-specific serine/threonine-protein kinase 2 (Kim *et al.* 2019) or ejaculatory bulb-specific protein (Liu *et al.* 2020) in the male stage, respectively. One cluster (cluster 14) was specific for both adult sexes but was enriched only for the carbohydrate metabolic process (GO:0005975). In contrast, cluster 9 (comprised of the pupa and both adult sexes) was enriched for several GO categories: cellular aa metabolic process (GO:0006520), catabolic process (GO:0009056), biosynthetic process (GO:0009058), and cellular nitrogen compound metabolic process (GO:0034641; see Figure 3 and Supplementary Table S10).

### Lepidopteran phylogenomics and detoxification gene content evolution

The phylogenomic analysis correctly placed *S. exigua* within the *Spodoptera* clade and as the sister-group to the clade containing *S. litura* and *S. frugiperda* (Figure 4; Le Ru *et al.* 2018; Kergoat *et al.* 2021). In addition, the inferred species relationships within Lepidoptera were in agreement with previous findings (Kawahara *et al.* 2019). We further scanned all lepidopteran genomes for gene families associated with detoxification functions. This included: gene families involved in phase I of the detoxification pathway such as cytochrome P450 and CCE (Kant *et al.* 2015); gene families involved in phase II, such as UGT and GST; and the gene family ABC involved in phase III (Li *et al.* 2007; Heidel-Fischer and Vogel 2015; Kant *et al.* 2015). Based on the annotation of the lepidopteran genomes, we searched for expanded detoxification-related genes (Figure 4 and Supplementary Table S16). Expansion of major genes families involved in detoxification was mainly visible for *S. frugiperda* (“corn” strain) within the Noctuidae. In the following, we analyzed in greater detail several lineage-specific genes.

### Potential lineage- and stage-specific candidate genes as targets for pest-control

We used OrthoFinder v. 2.3.11 (Emms and Kelly 2015) to identify homologous gene sequences in the genomes of eight closely related but diverse lepidopteran species, including three *Spodoptera* species, *S. exigua*, *S. litura*, and *S. frugiperda*. We aimed to identify *Spodoptera-*specific OGs, as such lineage-specific genes would be candidates for targeted pest-outbreak management development. We identified in total 119 OGs containing genes from only the three *Spodoptera* species (Supplementary Table S13.1).

Since the larval feeding stage of *Spodoptera* is the most detrimental to crops, we further selected seven OGs for which the *S. exigua* gene representative is DE in the larval stage cluster (cluster 4). For three of the seven genes, the closest homologs were “uncharacterized” proteins (Supplementary Table S13.2). The four remaining genes were annotated as: nuclear complex protein (OG0013351), REPAT46 (OG0014254), trypsin alkaline-c type protein (OG0014208), and mg7 (OG0014260; Supplementary Table S13.2). We confirmed the expression of all seven genes by checking the number of RNA-Seq reads mapped to each assembled transcript based on the results of the transcript abundance estimation with RSEM. The read count in the larval stages (first and third larval stages) was higher than in the other stages (Supplementary Table S17). Several reads derived from other stages mapped to the protein sequences, but the number of these mapped reads was low (Supplementary Table S17).

For the four putative lineage- and stage-specific annotated genes, we validated their *Spodoptera-*specificity by constructing gene trees of *Spodoptera* sequences with their most similar sequences identified from other lepidopteran species. We confirmed *Spodoptera*-specificity when all *Spodoptera* sequences in the gene tree reconstruction clustered together in a monophyletic group. For two of the annotated genes (mg7 and REPAT), we constructed two different gene trees. These gene trees were built on two different datasets (extended and reduced). The identification of putative homologs in related species varied per gene as well as the number of included sequences and species for the gene tree analyses [nuclear complex protein (OG0013351): 20 sequences, 3494 aa positions, REPAT46 (OG0014254) extended dataset containing both αREPAT and βREPAT clusters: 153 sequences, 863 aa positions, reduced dataset containing only the βREPAT cluster: 91 sequences, 717 aa positions, trypsin alkaline-c type protein (OG0014208): 69 sequences, 1101 aa positions, and mg7 (OG0014260): extended dataset: 27 sequences, 368 aa positions, reduced dataset: 17 sequences, 350 aa positions].

The gene tree of the nuclear pore complex proteins showed that the *Spodoptera-*specific genes form a single cluster, nested within lepidopteran DDB_G0274915-like nuclear pore complex proteins and sister to *Helicoverpa* sequences (Supplementary Figure S5)*.* The reduced mg7 dataset comprised sequences from the *Spodoptera*-specific OG in addition to the ortholog group “15970at7088” from OrthoDB. For the extended mg7 dataset, we additionally included all “mg” protein sequences according to He *et al.* (2012). The ortholog group “15970at7088” included nine single-copy genes present in other butterfly species and we found two paralogous copies in *S. litura*, likely due to a specific gene duplication. In order to evaluate whether other paralogs were present in any of the *Spodoptera* gene sets, we blasted the protein sequences against a local blast database of mg7 sequences comprising the sequences from OrthoDB, OG0014260, and He *et al.* (2012). In *S. exigua*, we identified three paralogs, which according to the GFF file are located (mRNAs) consecutively on the genome: 1268792–1275628, 1276053–1279376, 1280841–1286731. Similarly, in *S. litura*, we identified two and three paralogs in *S. frugiperda*. To test if the existence of multiple paralogs for mg7 is specific for *Spodoptera*, we analyzed the protein sets of five related Lepidoptera species as used in the initial OrthoFinder run. Running the same blast searches but using the protein sets of *B.* *mori*, *H.* *armigera*, *H.* *zea*, *H.* *virescens*, and *T.* *ni* all detected a single gene copy with reliable BLAST scores. Both the reduced and the extended mg7 gene trees included all identified *Spodoptera* paralogs. The reduced mg7 gene tree including all paralog *Spodoptera* genes and the single-copy homologs from OrthoDB showed that *Spodoptera-*specific OG sequences were clustered together (Supplementary Figure S6). This cluster formed a sister clade to all remaining *Spodoptera* paralogs and the *H. armigera* gene. In the extended mg7 gene tree, the *Spodoptera-*specific OG sequences did not form a monophyletic clade but did cluster together with the mg7 genes of *C.* *fumiferana*, *H. armigera*, and *S. litura* derived from He *et al.* (2012) (Supplementary Figure S7).

For the REPAT gene analyses, we compiled two datasets. Both datasets consisted of sequences derived from the *Spodoptera*-specific OG, the MBF2 ortholog group “16151at7088” from OrthoDB and all protein sequences according to Navarro-Cerrillo *et al.* (2013). The reduced dataset only contained protein sequences belonging to the βREPAT class, whereas the extended dataset included both αREPAT and βREPAT classes. In both gene tree analyses, the *Spodoptera-*specific OG sequences clustered together with the annotated REPAT46 gene from *S. exigua* (Supplementary Figures S8 and S9). The *Spodoptera-*specific OG is placed in the βREPAT cluster, sensu Navarro-Cerrillo *et al.* (2013), where it is placed within group VI (Navarro-Cerrillo *et al.* 2013). Further, in total 54 putative REPAT proteins have been identified in the *S. exigua* protein set which were included in both gene tree datasets (Supplementary Table S18).

The gene tree of the trypsin proteins showed a monophyletic clustering of all Lepidoptera-derived trypsin genes (Supplementary Figure S10). In addition, all *Spodoptera* trypsins were clustered within one monophyletic clade, with the *Spodoptera-*specific OG nested within. Trypsins occurred in all Lepidoptera species in large numbers, thus we compared various OrthoFinder runs under different stringency settings [varying the inflation parameter from 1, 1.2, 1.5 (default), 3.1, and 5] to test the degree of “*Spodoptera-*specificity” of this OG. In all five runs, the OG containing the *Spodoptera* trypsin genes was stable (*e.g.*, lineage-specific) and remained unchanged.

## Discussion

Using a combination of Oxford Nanopore long-read data and Illumina short-read data for the genome sequencing approach, we generated a high-quality genome and transcriptome of the beet armyworm, *S.* *exigua*. These resources will be beneficial for future research on *S. exigua* and other noctuid pest species. The developmental gene expression profile of *S. exigua* demonstrated that the transition from embryo to larva is the most dynamic period of the beet armyworm’s transcriptional activity. Within the larval stage the transcriptional activity was highly similar between early (first) and late (third) instars, making the early larval stage an ideal stage for pest-control (see below). Genes involved in the secondary metabolic process (GO:0019748) were only expressed in the larval stages (Figure 3). In addition, several prominent genes involved in digestion and detoxification, including cytochrome P450s and UGTs, and potential target genes for pest control could be identified which are specifically expressed in the larval stage (Supplementary Table S4).

The significant enrichment in the pupal stage in processes associated with anatomical structure development reflects the dramatic structural changes of the larva to the adult (Truman and Riddiford 2019). The identified pupal cuticle proteins within the pupal stage have been reported previously by other studies and reflect the morphological changes in wing disc and the larva-to-pupa metamorphosis (Gu *et al.* 2013; Ou *et al.* 2014).

The gene expression analyses of the developmental transcriptome of *S. exigua* revealed larval stage-specific upregulated genes (cluster 4, Figures 2 and 3). These identified genes are strong candidates for targeted RNAi of feeding larvae. Targeted RNAi of genes involved in vital functions of the most important larval stage can be an efficient strategy to minimize the detrimental effect of pest species (Xue *et al.* 2012). The larva stages of Noctuidae insects are the most damaging to plants. Our homology search revealed seven potential *Spodoptera*-specific genes with upregulation in the first- and third-instar larval stages, and highest expression levels in the third-instar stage (Supplementary Table S17). Four of these seven genes were annotated and we confirmed for three of them *Spodoptera*-specificity by gene tree analyses.

One putative *Spodoptera-*specific OG consisted of nuclear pore complex proteins. These proteins are involved in the transport of particles through the nuclear envelope (Alber *et al.* 2007). Although the gene tree did not follow well-established lepidopteran relationships (Kawahara *et al.* 2019), for example, Noctuoidea nested within Papilionoidea (Supplementary Figure S5), all identified *Spodoptera* nuclear pore complex proteins clustered together. This is a prerequisite for potential target genes, showing a clear separation of *Spodoptera-*derived sequences to sequences of other species.

We identified mg7 as a potential target gene for RNAi. This gene was previously reported to be highly upregulated in all larval stages in the midgut of *S. litura* with an expression peak after larvae have molted into the sixth larval stage (He *et al.* 2012)*.* Our results show a similar pattern with an increased expression towards the third-instar larva (Supplementary Table S17). Expression in the midgut suggests a role in digestion-related processes (He *et al.* 2012). Only the gene tree based on the reduced dataset showed clustering of *Spodoptera-*specific mg7 genes (Supplementary Figure S6). He *et al.* (2012) reported several homologs, mg2, mg7, mg9, and mg17 in related species which we included in the extended gene tree reconstruction (Supplementary Figure S7). The genes derived from the *Spodoptera-*specific OG form a monophyletic group with the mg7 genes of *C. fumiferana, H. armigera*, and *S. litura* derived from He *et al.* (2012), establishing orthology of Noctuidae and Tortricidae sequences and consequently challenging the *Spodoptera*-specificity for this candidate gene. The spruce budworm, *C. fumiferana* is a notorious conifer-feeding pest restricted to the Nearctic region where it is considered one of the most destructive insect defoliators (Lumley and Sperling 2010; Volney and Fleming 2007). The extended phylogeny identified homologous clusters (although with low support values) of “mg” genes (mg7, mg17, and mg9) in related lepidopteran species. The close relationship of additional gene family members from other lepidoptera makes mg7 more a potential candidate for RNAi-based pest-formation control in a wider range of lepidopteran pest species with the caveat that more work is needed to resolve lineage- and/or *Spodoptera*-specificity.

Finally, a strong potential target gene for biocontrol are the αREPAT proteins which are involved in various physiological processes and can be induced in response to infections, bacterial toxins and other microbial pathogens within the larval midgut (Herrero *et al.* 2007; Navarro-Cerrillo *et al.* 2013). Upregulation of REPAT genes has been identified in response to the entomopathogenic *Bacillus thuringiensis* (Herrero *et al.* 2007). In *S. frugiperda*, REPAT genes were associated with defense functions in other tissues than the midgut and found to be likely functionally diverse with roles in cell envelope structure, energy metabolism, transport, and binding (Machado *et al.* 2016).

REPAT genes are divided in two classes based on conserved domains. Homologous genes of the αREPAT class are identified in closely related *Spodoptera* and *Mamestra* species, whereas βREPAT class homologs are identified in distantly related species, for example, HMG176 in *H. armigera* and MBF2 in *B. mori* (Navarro-Cerrillo *et al.* 2013). Our analyses found that REPAT genes (and homologs like MBF2 members) from distantly related species are nested within the βREPAT cluster, while the αREPAT class is exclusive for *Spodoptera* and very closely related species like *Mamestra* spp. (Navarro-Cerrillo *et al.* 2013; Zhou *et al.* 2016; Supplementary Figures S8 and S9). In contrast to Navarro-Cerrillo *et al.* (2013) where αREPAT and βREPAT form sister clades, our tree topology show αREPAT genes to be nested within βREPAT.

Previously, 46 REPAT genes were reported for *S. exigua* (Navarro-Cerrillo *et al.* 2013), while we detected 54 REPAT genes in the *S. exigua* genome (Supplementary Table S18). The genes of *S. exigua*, *S. litura*, and *S. frugiperda* from the *Spodoptera-*specific OG as identified here cluster together with REPAT46 from *S. exigua* and thus are group VI βREPAT genes (Supplementary Figure S8). As shown in Navarro-Cerrillo *et al.* (2013) and here (Supplementary Figure S8), group VI βREPATs are comprised of *Spodoptera-* and other noctuid-derived genes, like *Helicoverpa* and *Mamestra*. The Noctuidae family is one of the most damaging groups of pests to agriculture, which is recognized by naming of a “pest clade” where species from the genera *Spodoptera*, *Helicoverpa*, and *Mamestra* are included (Mitchell *et al.* 2006; Regier *et al.* 2017). Overall, the results presented here show that REPAT gene members of especially the αREPAT class and the group VI βREPATs are putatively promising candidates for targeted RNAi in notorious pest species belonging to *Spodoptera* and closely related genera in Noctuidae, given their *Spodoptera-* and/or Noctuidae-specificity.

## Conclusions

The genome and developmental transcriptome including all major stages: embryonic, larval, pupal, and adult stages of both sexes, of the beet armyworm *S.* *exigua* provides a valuable genomic resource for this important pest species. Using a dual sequencing approach including long- and short-read data, we were able to provide a genome that is comparable to fellow lepidopterans, strongly supporting the use of these resources in further genome comparisons. Based on the differential gene expression analyses, we identified developmental stage-specific (embryonic, larva, pupa, or adult) or sex-specific (female, male adult) transcriptional profiles. Of particular interest are the identified genes specifically upregulated in the larval stages because those stages are most detrimental to the host plants. We have further validated these larva-specific genes for their suitability for RNAi-based targeted pest control by comparative genome analyses. RNAi-mediated insect control can be a powerful tool if selected target gene(s) are essential genes in insect tissues to trigger toxic effects. In addition, the target gene(s) should be pest species-specific or specific to a range of closely related pest species and should not harm nontarget organisms. In this context, *Spodoptera* lineage-specific target gene(s) are of high interest due to the high number of notorious pest species in this genus causing enormous agricultural damage resulting in economic losses worldwide. Analyzing the homologous relationships of the identified potential target genes and including a broad selection of other insect species allowed us to verify the specificity of three candidate genes for the genus *Spodoptera* and one candidate for RNAi-based pest-formation control in a wider range of lepidopteran pest species. Additional in-depth research may further confirm the clade-specificity of these genes and their potential application in RNAi-mediated pest-outbreak management.

## Data availability

The final genome assembly was submitted to the NCBI GenBank database and is available under the BioProject PRJNA623582, accession JACEFF000000000, version JACEFF010000000 is used in this study. All raw reads from the Illumina, MinION, and PromethION sequencing runs and Illumina RNA-Seq run were submitted to the NCBI SRA database under accession number PRJNA623582.

Supplemental material available at figshare: https://doi.org/10.25387/g3.14995326.

Further genome datasets and other datasets generated during the current study are provided at the Dryad digital repository https://doi.org/10.5061/dryad.280gb5mq6.
